# Effectiveness of the CATCH (Coordinated Approach to Child’s Health) Rainbow Program in Elementary Schools for Change in Fruit and Vegetable Intake

**DOI:** 10.3390/nu16193283

**Published:** 2024-09-27

**Authors:** Henna Muzaffar, Ashley Valinskas, Ashley Werner, Nora Collins, Melanie Regan

**Affiliations:** 1 School of Health Studies, Northern Illinois University, 209A Wirtz Hall, 370 Wirtz Drive, DeKalb, IL 60115, USA; 2 Rockford Public School District, 1212 E. Algonquin Road, Schaumburg, IL 60173, USA; rdashley.balance@gmail.com; 3 Rogers Behavioral Health, 9916 75th Street, Kenosha, WI 53142, USA; ashleywerner12@gmail.com; 4 Mount Sinai Hospital, 704 Stacie Court, Naperville, IL 60563, USA; 5 Nutrition, Dietetics & Wellness Department, Northern Illinois University, DeKalb, IL 60115, USA; z1884283@students.niu.edu

**Keywords:** cooking, gardening, fruits and vegetables, elementary schools, veggie meter, CATCH rainbow program

## Abstract

Background: Nutrition, cooking, and gardening lessons individually and together have been shown to increase fruit and vegetable (FV) consumption in school-aged children. The CATCH Rainbow program incorporated nutrition education, cooking, and gardening lessons aimed at increasing FV consumption in elementary school-aged children and assessed changes in participants’ BMI, self-reported FV consumption, and skin carotenoid levels at baseline and post-intervention. Methods: Two-hundred and twenty-five 4th and 5th graders (mean age: 9.8 years and 52% male participants) at Genoa Elementary School participated in six cooking and six gardening sessions between September 2021 and May 2022. Each nutrition education session was 25 min long, paired with either hands-on cooking activities or gardening skills. At baseline and post-intervention, participants’ height and weight were assessed with a stadiometer/scale, and skin carotenoid measurement was taken by a Veggie Meter^®^ (Longevity Link Corporation (Salt Lake City, UT, USA)). Students also completed the Block Food Frequency Questionnaire to self-report FV consumption at both time points. Focus groups were conducted with children at the end of the program for qualitative feedback. Results: paired samples *T*-test and regression analysis results indicate no significant decrease in BMI or significant increase in skin carotenoid scores from pre- to post-intervention. However, though not significant, there was an increase in self-reported FV intake by 0.4 servings. Additionally, the qualitative feedback was positive, as children mentioned benefits of healthy eating and expressed enjoyment for growing, cooking, and tasting fruits and vegetables. Conclusions: Results from this study can be used to guide future cooking and gardening programs for elementary school children. Time of the year when implementing these programs and collecting data may impact study outcomes due to seasonal variations in fruit and vegetable intake.

## 1. Introduction

A balanced diet is essential to health and wellness, as it provides nutrients, vitamins, and minerals the body needs for optimal function. Several benefits to healthy nutrition throughout the lifespan include reducing high blood pressure, lowering cholesterol, improving recovery times from injury or illness, improving immunity, and increasing overall energy levels [1]. Healthy eating patterns include following a well-balanced diet that provides all the necessary nutrients; this would include a diet encompassing whole grains, lean proteins, low-fat dairy, fruits, vegetables, and healthy fats [1,2,3]. Fruits and vegetables provide a vast array of vitamins and minerals that the body needs to work efficiently. There are possibly hundreds of micronutrients, phytochemicals, carotenoids, and different plant compounds found in the various fruit and vegetable families that are beneficial to health [3]. To list a few of the many, minerals such as sodium and potassium found in tomatoes and potatoes maintain fluid balance within the body, and vitamins such as vitamin A and K found in dark leafy greens help with physiological processes such as vision and bone health [3,4]. That is why eating a variety of fruits and vegetables from different families optimizes potential benefits from their nutritional properties. Currently, less than 50% of children in the US eat enough fruit to meet recommendations, and less than 12% eat enough vegetables [5]. In general, at least half of your plate should contain fruit and vegetables at each meal [2]. According to the Dietary Guidelines for Americans 2020–2025, the specific number of servings needed per day of fruit and vegetables is dependent on a person’s total calorie intake. For those eating a 1000–2000-calorie diet, fruit servings range from 1 to 2 ½ cups per day, and for vegetables, recommendations range from 1–2 cups per day [2].

Those who have a diet lacking in fruits and vegetables have higher risks for chronic diseases such as obesity, hypertension, coronary heart disease, stroke, and cancer [5]. Adults who are obese risk co-morbidities such as high blood pressure, high cholesterol, type 2 diabetes, asthma, sleep apnea, joint problems, cardiovascular disease, and more; for children who are obese, their risk for these health concerns in the future is also heightened [2,6,7,8,9]. According to the Centers for Disease Control and Prevention (CDC), one in five children and adolescents in the United States are affected by childhood obesity [10]. This demands attention and intervention to reduce the number of American children who are obese and risk potential complications in their adult life.

A person’s eating habits carry on from childhood to adulthood, which prompts nutrition interventions to happen earlier in life rather than later to prevent the risk of obesity and further diet-related chronic diseases [2,11]. Research has linked unhealthful eating habits to declined cognition; furthermore, these studies have linked poor nutritional quality to less success academically [12]. Researchers have indicated that children’s brain development is hindered by poor nutritional quality within their diets. This is usually a rapid process in early life and healthy nutrition is essential. Children who have a low-quality diet may have lower academic achievement. Poor academic achievement has additionally been linked to obesity, lower income, and even unemployment later in life [12]. In an Australian cohort study of 2287 participants, a 24 h diet recalls for children were collected at ages one, two, and three years old from their parents. Results showed that a higher-quality diet score at as early as age one led to higher scores in math, reading, and spelling when tested at age seven. They concluded that quality of early diet may be a predictor for later academic achievement [12]. This continues past ages one, two, or three, and is true throughout a child’s school-age years, emphasizing the importance of a balanced diet throughout the lifespan. For precisely this reason, early nutrition intervention is key to a child’s development and success later in life. The earlier a person is exposed to fruits and vegetables and develops healthy eating habits, the more likely they are to carry on those habits in the future [2,12].

There is a lack of effective nutrition interventions in society today from childhood to older adulthood, as there is no current standard method of teaching nutrition information to different age groups. The school environment is one of the most influential places for children to learn about nutrition and be exposed to new foods [11]. Students receive anywhere from one-third to half of their nutrition for the day from their school cafeteria [13]. Researchers and health professionals have been experimenting with various interventions and studying their impact on FV intake in elementary aged school children. Methods of interventions range from nutrition lessons through videos, lectures, and worksheets to cooking demonstrations, taste tests, hands-on cooking lessons, gardening lessons, or any combination of the above listed. The search for an effective, engaging program is ongoing and seems to depend on the age group, demographic area, and resources for nutrition education/interventions within the school.

Nutrition education lessons have proven to be impactful on students’ self-efficacy, knowledge, and preference towards fruits and vegetables [14,15,16,17]. Beyond lessons in the classroom, other studies have shown that hands-on approaches are more effective. The “Students Pick a Better Snack” study conducted in Las Vegas provided students with nine monthly nutrition education lessons and cooking demonstrations with taste tests lead by a trained chef/registered dietitian (RD). Research discovered that the intervention group (n = 380) had improved their attitude towards fruits and vegetables when compared to the control group (n = 99) [17]. Cooking lessons are a popular way to expose children to new foods and healthier choices early on in life. Providing students with gardening lessons has been another area of nutrition interventions in which students have shown interest and modeled behavior change. A study published in 2020 by Kim and Park included a 12-week intervention where 202 elementary school students participated in nutrition education, gardening lessons, and cooking lessons that used the garden’s harvest [18]. Pre- and post-program evaluation questionnaires were collected from the children. They found that dietary self-efficacy, outcome expectancies, gardening knowledge, nutrition knowledge, vegetable preference, and vegetable consumption were significantly increased, and food neophobia was significantly decreased [18]. Research suggests that nutrition education can positively influence nutrition knowledge, whereas hands-on lessons can provide students with life skills to adopt healthy habits [18]. In addition to improving FV intake, cooking and gardening lessons also increase the consumption of unprocessed foods and decrease the consumption of ultra-processed foods [19]. Programs that focus on gardening and include a tasting component have shown to be more successful than gardening interventions alone [6,20]. These combined gardening and tasting programs have demonstrated improvement in students’ thoughts and feelings towards vegetables and healthy choices, as seen for cooking programs [6,20]. Nine schools from Wisconsin participated in a study observing the effectiveness of a Farm-to-School program. In this program, students (N = 1117) took gardening lessons, tended to their school garden, and taste tested the produce. Results showed an increase in overall attitudes towards vegetables, vegetable knowledge, exposure, and willingness to try new things [20].

The Coordinated Approach to Child Health (CATCH) program is a school-based nutrition education program using age appropriate content to improve nutrition and physical activity behaviors of children by promoting healthy eating, increasing physical activity, and reducing screen time to combat childhood obesity [21]. The CATCH Rainbow program is based on the CATCH nutrition education curriculum, with additional lessons focusing on cooking and gardening activities. The purpose of this study is to evaluate the effectiveness of implementing weekly cooking and gardening lessons to fourth and fifth graders at Genoa Elementary School on body mass index (BMI), and fruit and vegetable consumption, as measured by the Veggie Meter^®^ (skin carotenoid scanner) scores and Food Frequency Questionnaire (FFQ) administered before and after participation in the program. The primary aim was to increase fruit and vegetable consumption in school-aged children by eliciting interest and familiarity to various fruits and vegetables through basic cooking, taste testing, and gardening activities. Researchers also sought qualitative feedback from the participants by conducting focus groups after the program ended.

## 2. Methods

**Figure 1:**
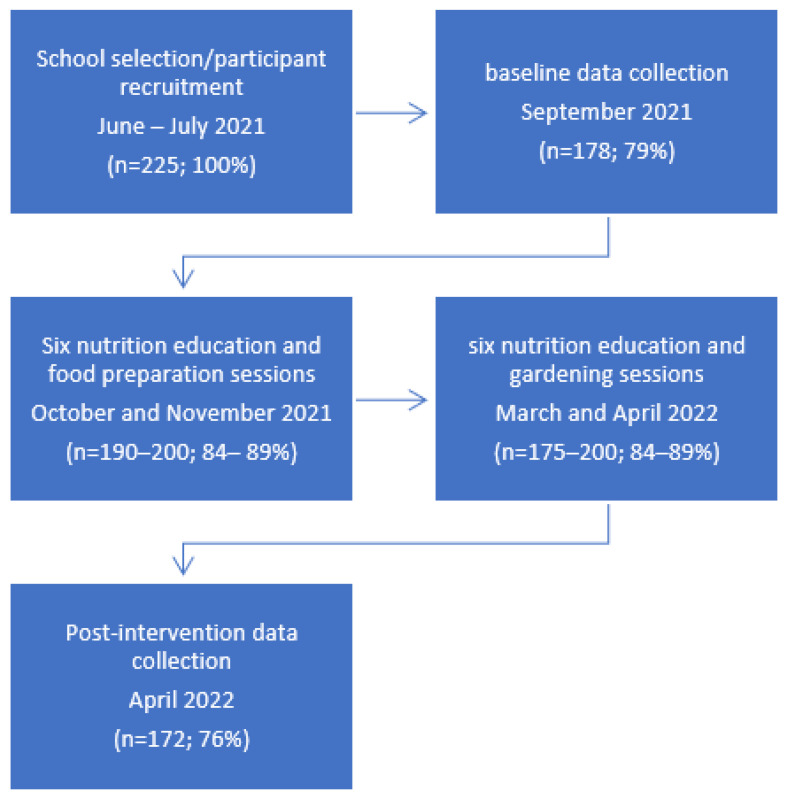
Flowchart depicting the five stages of CATCH Rainbow program.

The CATCH Rainbow program was implemented as a pilot study at Genoa Elementary School in Illinois from September 2021–May 2022. As this was a pilot study, the program was only delivered in one school. The principal investigator (university professor) reached out to five elementary schools in Northern Illinois, and Genoa Elementary School was the first school to indicate their interest in receiving the CATCH Rainbow program. The university professor and her four graduate students made 15 visits (baseline data collection visit, 12 program lessons, post-intervention data collection visit, and focus group visit) to the school for data collection and program implementation. Six cooking/tasting sessions were delivered once a week for 6 weeks in October and November 2021, and six gardening sessions were delivered once a week for 6 weeks in March and April 2022. The lessons included mini-lectures, videos, worksheets, and interactive activities. Students were given a chance to interact with their peers in the same section for completing worksheets and participating in cooking and gardening interactive activities. To ensure consistency in program implementation and data collection, the university professor trained the graduate students for data collection procedures and program methods. The program instruction occurred during the school day and 100% of the program content was taught in each classroom. All five research team members were present at each visit and each section received the same lesson on the same day. Institutional Review Board (IRB) approval was obtained for this study from the Office of Research Compliance, Integrity & Safety at Northern Illinois University (Protocol Number: HS21-0457). A passive informed consent process was used to recruit participants in this program. A detailed informed consent was emailed to the parents, which provided information about the program and the data collection measurements. The consent form also included contact information for the university professor in case the parents wanted to reach out with any questions or concerns. If any parent, after reading the program details, did not want their child to participate in the program, they were asked to decline participation on the consent form, sign the form, and send the form back to the school (a passive consent process is when the parents receive detailed information about the study procedures and data collection tools, and the parents sign and return the form if they do not want their child to participate or do not want any data to be collected on their child.) Five parents returned the consent form, indicating that they do not want their child to participate in data collection and no data were collected from those children. A flowchart (Figure 1) below depicts the process through the five stages of recruitment, data collection, and program implementation.

### 2.1. Study Population

Two hundred and twenty-five 4th and 5th graders from Genoa Elementary School participated in this research study. There were five 5th grade classrooms and four 4th grade classrooms with an average of 25 students (range 23–29 students) per class who participated in the program. In the initial data collection phase, 178 students completed the baseline data collection. During the post-intervention data collection phase, 172 students completed the forms. Attendance was taken at each of the lessons and 90% of the students in each class attended each of the program sessions, as the attendance rate at this school is 90% of the students present in the school on any given day and 10% absent due to a variety of reasons.

### 2.2. Recruitment

In April 2021, to identify one elementary school to receive the CATCH Rainbow program, elementary schools in the area surrounding Dekalb, IL were emailed about potential interest in participating in the CATCH Rainbow program. Genoa Elementary School responded saying that they would be willing to participate. More information about the program and who it involves was given to the principal. Once he agreed, a passive consent form was sent out to the parents via an email. The two inclusion criteria for this program were students in 4th or 5th grade at the selected school and the parent’s consent via passive consent process. The passive consent strategy for this study was approved by the IRB at Northern Illinois University.

### 2.3. Data Collection and Description of Instruments

Data were collected from the participants at both baseline and post-intervention. Data collection included quantitative, demographic, anthropometric, and post-qualitative data. Quantitative data were obtained from the Food Frequency Questionnaire (FFQ) with 77 food and beverage questions, including frequency and portion size. Demographic data included gender, age, race, and ethnicity. Anthropometric data included the Veggie Meter^®^ score, height (cm), weight (lbs.), and BMI percentile (obtained from collected height and weight). Post-qualitative data included responses recorded from the nine focus groups. Below, please find the details for all the data collection tools used in this study.

#### 2.3.1. Demographics Form

The demographics form included information about age, gender, height (cm), weight (lbs.), and the ethnicity of the participant. Participants were assigned an identification number printed on the demographics form and then matched with the identification number on their FFQ. A combined stadiometer and scale were used to obtain height and weight measurements to the nearest 0.1 cm and 0.1 kg, respectively (Seca Portable Stadiometer combinable with Flat Scale (Chino, CA, USA, 91710)) in a private corner in the room. A university researcher had the participant step onto the scale and then the researcher read and recorded their height and weight onto the demographics form. The height and weight information were then used to calculate body mass index (BMI) percentile-for-age using the guidelines from the Centers for Disease Control and Prevention (CDC) [22,23].

#### 2.3.2. Carotenoid Skin Scanner (Veggie Meter^®^)

The Veggie Meter^®^ is a widely accepted and validated tool to assess fruit and vegetable intake [24,25,26,27]. The Veggie Meter^®^ is manufactured by Longevity Link Corporation (Salt Lake City, UT, USA), a company that creates technology for detecting micronutrients in living human tissue [28]. The Veggie Meter^®^ uses reflective spectroscopy as a non-invasive way to measure skin carotenoid levels which correlate with dietary carotenoid intake. The scanner works by emitting a white LED light through the fingertip. Carotenoids in the skin have a characteristic absorption band in the blue wavelength of the visible light spectrum. The absorption seen by the scanner directly correlates to the concentration of carotenoids present in the skin. Before having the participant place their finger into the scanner, the researcher explained the Veggie Meter^®^ process to the participants to reduce any apprehension about the scanner [29]. The researcher wiped the participants’ index finger clean using a disinfectant wipe and then placed their finger into the scanner. The Veggie Meter^®^ asks for the participant’s gender, height, and weight to calculate an accurate score. Scores can range from 0 to 800, with a higher skin carotenoid score indicating higher intake of fruits and vegetables.

#### 2.3.3. Block 2004 Food Frequency Questionnaire (FFQ)

The 2004 Block Food Frequency Questionnaire (FFQ) developed by Nutrition Quest makes for a suitable tool for this study, as it includes a wide range of food items and was designed for the age range (8–17 years) that participated in the CATCH Rainbow program [30]. The food items included in the survey were adapted from NHANES 1999–2002 dietary recall data [30]. A systematic review examining the different dietary assessment tools for children 11 years and younger reported that FFQ’s may be the best method for assessing dietary intake for this age range [31]. The current study aims to examine the effect of the program on overall FV intake before and after the intervention. This was accomplished by comparing the questionnaires’ total fruit and total vegetable serving estimates at baseline and post-intervention.

The FFQ asks participants about the types of foods they eat based on food group category, the frequency of consumption (how many times per week), and the portion size they normally consume. The Block FFQ used in this study included seventy-seven food items. The participants took about 25 min to complete the questionnaire on their own and asked the university researchers any questions if they needed help. For each food item, the survey asked about the frequency and portion size for that food item. For example, one item read, “Pancakes, waffles, pop tarts” and the respondent selected how many days they consumed that item last week. The response options were none, 1 day, 2 days, 3–4 days, 5–6 days, or every day. Next, the survey asked, “How many?” The options were ½, 1, 2, or 3. For some questions in the “How many?” category, the respondent referred to the portion size/servings page, where they were presented with different-sized bowls filled with different amounts of an example item, and the respondent picked A, B, C, or D. Individual portion size was asked, and pictures were provided to enhance the accuracy of the quantification.

#### 2.3.4. Focus Groups

**Figure 2:**
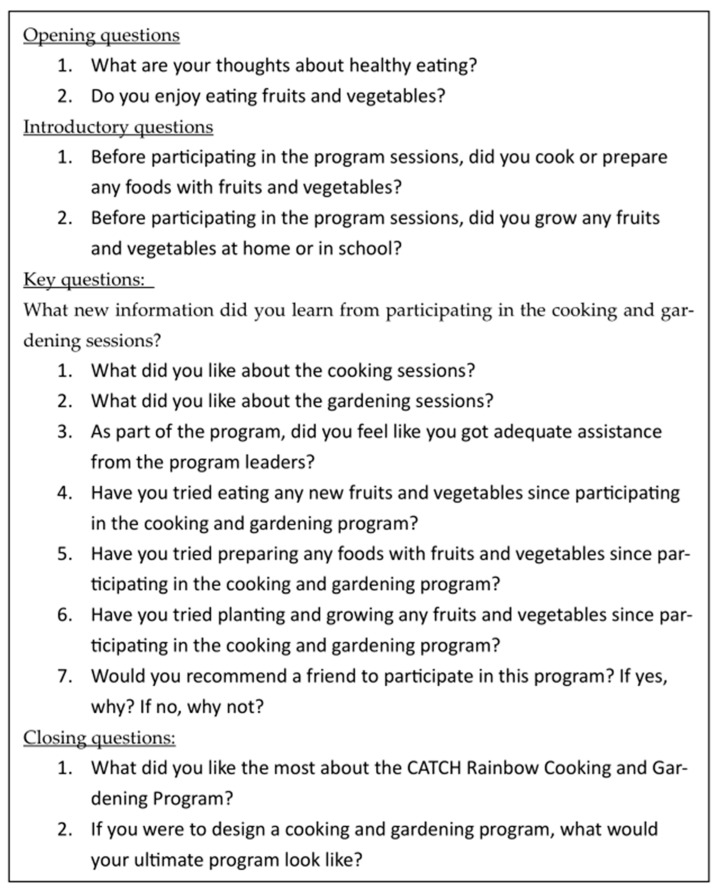
Focus group script for CATCH Rainbow cooking and gardening.

After the implementation of the CATCH Rainbow program, nine focus groups were conducted with a small subset of students from each section of the 4th and 5th grades who had participated in the program. In the focus groups, two boys and two girls, who were randomly selected by the classroom teacher, were asked to meet with the university professor and one university student in a separate room in the school. During each focus group, students were asked a series of questions that were predetermined by the professor and the university student. Participants were able to answer the questions one at a time, encouraged to share their thoughts and respond to their peers. The university student took notes during the focus groups and recorded the audio on their cell phone, which was then transcribed verbatim. Each focus group took approximately 15–20 min to complete. Students were then returned to their classrooms. The focus group included 14 questions, including opening, introductory, key, and closing questions, as shown in Figure 2.

### 2.4. Intervention

This study had two hundred and twenty-five 4th and 5th grade participants from nine different class sections. During the first visit in each classroom, four researchers introduced themselves, collected baseline data from the students, and gave them an overview of the cooking and gardening lessons. After the initial data collection visit, six nutrition education and cooking lessons were delivered to the students in the following six visits. Each lesson was 25 min in duration to fit in one class period for fourth and fifth graders and was delivered by the university professor and her students. The classroom teachers only assisted if any disciplinary issues arose during the lesson. Each cooking lesson talked about a part of the plant and was accompanied by a snack preparation. Students were also given a newsletter at the end of each lesson to take home to their guardian. The newsletter contained a summary of the lesson for the day and a recipe for the snack they had just enjoyed. The cooking lessons for the students were as follows:

1. The Seed: students were taught about the parts of the seed and what they do; accompanied by a trail mix with chickpeas.
2. The Root: students learned where roots are found and their function within the plant; accompanied by a beet apple salad.
3. The Stalks and Stem: students learned the function of the stem; accompanied by ants on a log snack.
4. The Leaves: students examined the different texture, taste, look, and smell of leaves; accompanied by three different leaves and salad dressing snack.
5. The Flowers: students examined the growth of flowers and how they bloom; accompanied by a smoothie that contained broccoli.
6. The Fruit: students learned how a plant makes fruit; accompanied by build-your-own fruit pizzas.

Following the six cooking lessons, six nutrition education and gardening lessons were then delivered in the spring semester. These lessons were also 25 min in duration and were taught by the university professor and her students. These six lessons were designed to teach students about gardening and how to plan their own garden, leading up to the implementation of a school garden on their school property. Students were also given a newsletter at the end of each lesson to take home to their guardian. The newsletter contained a summary of the lesson for the day and ideas for gardening activities at home. The gardening lessons were as follows:

1. Design Your Garden: students were given a worksheet to design their own garden and pick what plants they might want to grow.
2. Companion Planting: students learned what companion planting means and which plants are friends and enemies.
3. Sowing Seeds Indoors: students were given seeds, egg cartons, and dirt; students planted their seeds in these cartons and were informed when to water them.
4. Transplant and Garden Action Plan Part 1: students transferred their plants from the egg cartons to larger plastic-colored cups.
5. Garden Action Plan and When to Harvest: students discovered the various times plants needs to grow, when they should be planted, and when they are ready to be harvested.
6. Transplant Day: Students brought their plants in the plastic-colored cups outdoors where they were planted in the outdoor garden.

After the full delivery of the programs, researchers made a visit to collect data for post-intervention evaluation. One last visit was made by a university professor and one university student researcher who conducted nine focus groups with the children to obtain qualitative feedback.

### 2.5. Outcome Measures/Statistical Analysis

The data collected were used to assess the impact of the CATCH Rainbow program on three program outcomes: fruit and vegetable intake, anthropometrics, and appeal and acceptability of the program. Fruit and vegetable intake was the main outcome of this research study. The Veggie Meter^®^ scores and the results from the FFQ contributed to the evaluation of fruit and vegetable intake pre- and post-program implementation. BMI and BMI percentile were calculated from the anthropometric information collected from the participants. In addition, qualitative acceptability of the program was assessed by the focus groups. IBM SPSS Statistical package 26.0 was used to compute the results of this study (IBM Corp., Armonk, NY, USA). The following variables were recorded into an excel spreadsheet and then exported into the SPSS software (IBM Corp. Released 2021. IBM SPSS Statistics for Windows, Version 28.0. IBM Corp., Armonk, NY, USA): participant ID, height, weight, age, grade, gender, race, ethnicity, BMI, BMI percentile, and skin carotenoid score 1 and 2. FFQ results were analyzed by Nutrition Quest (https://www.nutritionquest.com/assessment/ (accessed on 15 March 2023)). Those results were then inserted into SPSS.

Descriptive statistics and frequencies were used to evaluate the demographic data from the study participants. Paired samples t-test was used to compare the means and test the statistical significance of change in VM scores and FFQ results from baseline to post-intervention. Regression analysis was used to test the results of the VM scores and FFQ results for predictability using age, gender, race, and ethnicity as potential moderators.

Grounded theory analysis was used to analyze the qualitative data from the focus groups conducted with the children participants. The transcribed responses were coded independently by two researchers, who then met in the presence of a third researcher to reach agreement on coding. The agreed upon codes were then coalesced to extrapolate themes that best represent participants’ experience with the program.

## 3. Results

### 3.1. Demographics

A total of 225 students were recruited for this study, as the participating school has nine sections of fourth and fifth graders with 23–29 students in each section. However, 199 students (88.4%) were present at the first session, out of which 178 students (79.1%) filled out the demographic data form and completed the food frequency questionnaire (Table 1). The study population included fourth (n = 83, 46.6%) and fifth (n = 95, 53.4%) graders. The ages of the students were nine (n = 52, 29.2%), ten (n = 87, 48.9%), and eleven (n = 15, 8.4%), and 13.5% (n = 24) participants did not report their age. Gender was reported as male (n = 92, 51.7%) or female (n = 80, 44.9%), and 3.4% (n = 6) participants did not report their gender (n = 6, 3.4%). Race and ethnicity were separated into two categories. The students reported their race as white (n = 33, 74.7%), African American (n = 6, 3.4%), or other (n = 22, 12.4%), with 9.6% of students not reporting their race. Ethnicity of the participants was reported as Hispanic (n = 31, 17.4%) or Non-Hispanic (n = 132, 74,2%), and 8.4% did not report their ethnicity. Lastly, BMI was recorded at the time of baseline data collection, with the average BMI for all participants being 20.356 and the average BMI percentile of 73.70%, indicating healthy weight [2].

### 3.2. Quantitative Findings

Of the 225 students recruited for this study, 159 (70.6%) students’ baseline skin carotenoid scores were recorded correctly, and 164 (72.8%) students’ post-intervention skin carotenoid scores were recorded correctly. The average skin carotenoid scores at baseline and post-intervention were 216.67 and 162.82, respectively. Students’ average Veggie Meter^®^ (VM) scores decreased by 54 points when comparing baseline and post-intervention scores (Table 2). This decrease was statistically significant (*p* < 0.01). Only the results from students who completed both the baseline and post-intervention FFQ were considered for estimating FV change (n = 168, 74.6%). The average intake of FV servings at baseline and post-intervention were 3.15 and 3.55, respectively. FV servings increased by an average of 0.4 servings between baseline and post-intervention, although not statistically significant (*p* = 0.075) (Table 2). BMI and BMI percentile were also calculated for participants at baseline and post-intervention. Table 2 also shows the comparison for BMI and BMI percentile between baseline and post-intervention.

A multiple regression analysis was run to predict VM scores with grade, age, gender, race, and ethnicity as potential moderators. The results showed that these variables, except one variable, do not predict VM scores significantly, F (5, 130) = 2.653, *p* = 0.026, R2 = 0.093. The only individual variable that showed statistical significance to predict VM scores was gender, *p* < 0.05. A second multiple regression analysis was run to predict FFQ results with grade, age, gender, race, and ethnicity as potential moderators. The results showed that these variables do not predict FFQ results significantly, F (5, 133) = 1.084, *p* = 0.372, R2 = 0.039.

The Pearson correlation test was used to assess the correlation between baseline and post-intervention VM scores and the FFQ results. Pre- and post-VM scores showed a significant moderate correlation (r = 0.530, *p* < 0.001). Pre- and post-FFQ results showed a non-significant moderate correlation (r = 0.596, *p* = 0.075). Correlation between the VM scores and FFQ at baseline was tested revealing weak correlation between the results (0.1 < |r| < 0.3), although not significant, as shown by the *p*-value of 0.313. The post-intervention scores from the VM and FFQ results exhibited a moderate correlation (0.3 < |r| < 0.5), although not significant, as shown by the *p*-value of 0.298.

### 3.3. Qualitative Findings

The results from the focus groups using grounded theory analysis revealed that students were enthusiastically in support of cooking and gardening programs. There were four major themes that emerged from the focus groups: benefits of healthy eating, cooking and tasting fun, joy of growing fruits and vegetables, and ultimate cooking and gardening program. Select quotes were pulled from various focus groups to reinforce these themes, which can be seen in Table 3. Students shared their thoughts on healthy eating and what it means to them. They discussed with researchers and each other about trying new foods they would not have at home or trying these fruits and vegetables in new ways. They also talked about how it felt to grow some fruits and vegetables at their school. Lastly, they focused on what their ultimate cooking and gardening program would look like and shared suggestions with the researchers (Table 3).

## 4. Discussion

The CATCH Rainbow program was implemented between September 2021 and April 2022 to elementary school children and incorporated nutrition education, cooking lessons, gardening sessions, and parent newsletters. The primary objective of this study was to pilot test the effectiveness of the CATCH Rainbow program in increasing fruit and vegetable intake for fourth and fifth graders at Genoa Elementary School using objective and subjective data. The secondary objectives were to assess changes in BMI and BMI percentile, and to obtain qualitative feedback about the program via focus groups. The results indicate a significant decrease in VM scores, a non-significant increase in FV servings as indicated by the FFQ results, a significant increase in BMI and BMI percentile, and a favorable perception and feedback about the program.

Children in the US do not eat enough fruits and vegetables, prompting the need for effective interventions to increase their intake [5,32]. Based on the objective measurement of skin carotenoid status, there was a statistically significant decrease in VM scores by an average of −141.153 (*p* < 0.001), which was unanticipated by the researchers. This is unlike other studies that have used the Veggie Meter^®^ to test FV changes after program implementation [33,34]. In the studies conducted by Jones et al. and Obana et al., VM scores increased after nutrition education sessions [33,34]. In the study by Obana et al., they used the readings of the VM at baseline to educate students on how much FV they were currently eating and encouraged them to increase their intake by the next reading interval. They took measurements at baseline, and then 3-months and 6-months post-intervention. They saw an overall increase of 47.4 points from baseline to 6-months post-intervention [34]. The researchers concluded that the Veggie Meter^®^ was an effective educational tool to inform and motivate participants to increase their FV intake. In future studies, sharing the VM scores with the participants to educate and motivate children could be an effective strategy to promote FV intake. In the CATCH Rainbow program, we did not share the VM scores with the participants and did not use them as a motivational tool to promote FV intake. The decrease in VM scores from baseline to post-intervention in the current study is also unlike other research that observed positive FV intake changes using Healthy Eating Index 2015 scores as an evaluation metric after combined cooking and gardening programs [35,36]. The two Sprouts Program studies suggested overall positive dietary changes after implementing nutrition education, cooking, and gardening lessons using Healthy Eating Index 2015 scores [29,35].

Our results are similar to the study conducted by Beccarrelli et al., which used the FFQ and skin carotenoid scores assessed by resonance Ramen spectroscopy (RRS) to examine changes in FV intake before and after program implementation [37]. Similar to our findings, their study revealed a significant decrease in skin carotenoid levels (*p* = 0.04) [37]. There are several possibilities for why the scores decreased instead of increasing in our study, the first being the timing of program implementation. When baseline scores were taken, it was in September 2021, and post-intervention scores were taken in early April. The spring-to-fall timeframe is when fruits and vegetables are most available in this region, making it more likely that students would be consuming more fresh produce prior to baseline data collection. Post-intervention scores were taken in early spring, right after the winter months when the variety of produce in the Midwest is limited and includes the holiday season when people may be eating more treat foods rather than fruits and vegetables. The second reason could be that we took our VM reading on the index finger and were only able to take one reading for each participant due to time constraints. Two studies have suggested using participants’ non-dominant ring finger for scans to avoid calluses or marks that typically form on dominant hand digits [33,37]. Moreover, we were only able to take single Veggie Meter^®^ reading for each participant at pre- and post-intervention, when the standard of practice is to take three readings and use the average of those readings [29].

The FFQ results showed an average increase in FV intake by 0.4 servings, although this change was not statistically significant (*p* = 0.075). The results from this study are unlike the Bontrager Yoder et al. study, which used an FFQ to assess the effectiveness of a Farm-to-School program. Their study showed an increase in attitudes towards FV but there was no effect on overall dietary patterns when assessed by their FFQ results [19]. Other studies looking at the impact of FV intake after nutrition intervention have shown similar results in increasing FV intake [35,36].

The BMI and BMI percentile results indicate an increase in these measurements as opposed to the study hypothesis that the program participants would show improvement in their BMI percentile after participation in the CATCH Rainbow program. Research evidence is more conclusive in adults for increase in FV intake leading to reduction in BMI, whereas the studies with children have shown mixed results for increase in FV intake to reduce obesity parameters in children [38,39]. Our results are similar to the study conducted by Davis et al., in that the study participants increased their vegetable intake but did not decrease weight measures after participating in nutrition education and cooking and gardening lessons [39]. In fact, a meta-analysis of 19 school-based RCTs indicated that these programs were not effective at decreasing BMI in the intervention group compared to the control group. Another possible explanation for school-based programs not having significant improvements on weight outcomes is that BMI changes take a longer time than the dietary behavior change itself and most programs lack long-term and follow-up data collection from the participants. We did not evaluate our participants for improvement in any outcome variables after the post-intervention data collection. Programs that target overweight and obese children have more success in significantly improving BMI percentile [40]. In the CATCH rainbow program, the average BMI and BMI percentile was in the normal range at the time of baseline measurement.

The results from our study showed weak to moderate non-significant correlations between the use of the FFQ and VM. To our knowledge, this is the first study to test the correlation between these two instruments. The FFQ and Veggie Meter^®^ have both been separately tested for correlations to other tools in other studies [25,29,31]. This study shows the FFQ and Veggie Meter^®^ to be weakly correlated and indicates that more measurement intervals and additional tools could be used for better assessment and correlations in future studies [31].

The focus groups conducted by the researchers showed the support of the students for the CATCH Rainbow program through the themes identified. Students learned about healthy eating, stating that they understood the importance of eating healthy. They expressed their enjoyment for trying new foods, stating that they would not have tried them outside of the classroom. The students displayed a positive sense of responsibility to the plants they grew in school. Lastly, the students provided suggestions for future school nutrition education programs including tasting different foods from around the world. This study was like other research studies that assessed the acceptability of nutrition programs by seeking qualitative feedback from the students [41,42]. In fact, Lukas et al. used focus groups to reveal student perspectives that could strengthen the content of intervention programs [41]. Our study has a similar impact, as the results can inform changes for future programs based on students’ responses to focus groups. More studies need to be conducted testing the effectiveness of nutrition education programs in increasing fruit and vegetable intake [6,9,19,32,35].

### 4.1. Limitations

There were several limitations to this study that may have impacted the results of the intervention. One limitation of this study was that the program was only implemented in one school. All students were from the same town/area, limiting the applicability to the general population. Future research should include multiple schools’ participation from different areas with fewer time constraints. Second, there was limited classroom time for data collection and nutrition education lessons. Limited time for data collection could have increased human error when recording the data, especially the VM readings. Third, the Veggie Meter^®^ manual suggests taking an average of three scores for each participant. However, due to limited time, only one skin carotenoid score was measured for each participant. Fourth, several students expressed confusion and needed help filling out the FFQ form, which was unanticipated by the researchers. This may have caused some students to rush through the survey, impacting the quality of the data collected. Fifth, we did not collect 3- or 6-month follow-up data from our participants, which could have indicated improvement in VM scores, FV intake scores, BMI, and BMI percentile. Sixth, we did not assess any psychosocial mediators (knowledge, attitudes, preferences, or self-efficacy) related to FV intake, cooking skills, or gardening skills in this study, which show improvement before the behavior change itself and could have indicated the potential for the effectiveness of the CATCH Rainbow program in improving FV intake [43,44]. Seventh, due to COVID-19, the original intention of including participants in hands-on cooking activities was omitted, and the recipes were prepared by the university students in the classroom. It is possible that having students participate in the cooking lessons with a hands-on approach may have a more positive result on FV intake. Lastly, there was limited parent involvement for this intervention. Students were given the weekly newsletter to share with their parents. There was no other contact with parents/guardians or the participants’ families. Parents are a challenging population to reach, as studies that have included parent lessons have had a low attendance rate (10–30%). More research is needed to identify the best platforms to engage parents in school-based interventions [39,43].

### 4.2. Strengths

The strengths of this program include its multi-component approach to nutrition intervention. This program included nutrition education lessons, cooking lessons, gardening lessons, and a take-home newsletter for guardians. This study also used multiple types of data collection, including subjective and objective data from the FFQ, VM, and focus groups. This study provides both quantitative and qualitative data to test the effectiveness of the CATCH Rainbow program in increasing fruit and vegetable intake in elementary school-aged children. This study also provides insight for future researchers to develop and implement health promotion programs in schools. Lukas et al. stated that the utilization of student focus groups for programmatic feedback can help strengthen the content delivered during nutrition intervention programs as well as enhance the future implementation of such programs [41].

## 5. Conclusions

This study tested the effectiveness of the CATCH Rainbow program in increasing fruit and vegetable consumption in fourth and fifth graders at a local elementary school. The objective findings of the Veggie Meter^®^ scores showed a significant decrease in FV intake, while the FFQ results showed a non-significant increase in FV servings from baseline to post-intervention. The focus groups assessing the acceptability of the program indicated positive results in terms of change in knowledge, attitudes, skills, and behavior from participating in the combined cooking and gardening nutrition intervention. Future studies are indicated to include longer-duration multi-component nutrition intervention programs with elementary school-aged children, use of both quantitative and qualitative data collection for testing the program’s effectiveness for increasing FV intake, use of VM scores as a teaching tool to motivate children to increase their FV intake, assessing psychosocial mediators of behavior change, and follow-up data collection at 6-months or 1-year post-intervention to assess the maintenance of desired behavior changes.

## Figures and Tables

**Table 1:** Baseline demographics of CATCH Rainbow participants.

Characteristic		Frequency (%)
Grade	4th graders	83 (46.6%)
	5th graders	95 (53.4%)
Total		178 (100%)
Age	9	52 (29.2%)
	10	87 (48.9%)
	11	15 (8.4%)
	Did not report	24 (13.5%)
Total		178 (100%)
Gender	Male	92 (51.7%)
	Female	80 (44.9%)
	Did not report	6 (3.4%)
Total		178 (100%)
Race	White	133 (74.7%)
	African American	6 (3.4%)
	Other	22 (12.4%)
	Did not report	17 (9.6%)
Total		178 (100%)
Ethnicity	Hispanic	31 (17.4%)
	Non-Hispanic	132 (74.2%)
	Did not report	15 (8.4%)
Total		178 (100%)

**Table 2:** Pre- and post-intervention comparison for BMI, BMI percentile, carotenoid score, and FV servings.

	Baseline			Post-Test			
	N	Mean	SD	N	Mean	SD	Sig. (2-Tailed)
BMI (kg/m^2^)	172	20.356	4.8356	172	21.170	5.1127	<0.01
BMI percentile	172	73.70	26.781	171	76.24	25.652	0.002
Carotenoid score	159	216.67	83.571	164	162.82	79.796	<0.01
FV servings	168	3.1533	2.96825	168	3.5542	3.41505	0.075

**Table 3:** Themes revealed from focus groups.

Themes	Quotes
Benefits of Health Eating	“Healthy eating is important and it can be really good. It’s a lot of food that’s actually super healthy for you, and is probably some of their favorite foods,” (4th grader)
	“Good idea to help people get more energized,” (5th grader)
Cooking and Tasting Fun	“I liked that there was a lot of things we got to try, and it was really creative,” (4th grader)
	“It was good to see different foods and get to taste them even though not all of them satisfied my taste buds,” (4th grader)
Joy of Growing Fruits and Vegetables	“[it was] fun to learn how plants grow and how they survive and breathe and do all the stuff that humans do like eat, drink, breathe, and stuff like that,” (5th grader)
	“It was really cool to be able to take care of plants by ourselves, [and] us being responsible to do it,” (4th grader)
Ultimate Cooking and Gardening Program	“I would say go around the world grabbing and taking all different fruits and vegetables and maybe picking up new recipes and giving people an idea of what they can make at home,” (5th grader)
	“It helped us to eat healthier, to be more active, and to plant more plants, and that it was a program that actually truly helped us,” (4th grader)

## Data Availability

The data presented in this study may be provided on request from the corresponding author once IRB approval and appropriate data use agreements have been obtained.
